# Modified extrapedicular kyphoplasty for the treatment of lumbar compression fracture: Case report and technical note

**DOI:** 10.1097/MD.0000000000019053

**Published:** 2020-02-07

**Authors:** Li-Min Wang, Feng-Yu Liu, Kuan Lu, Zhao Liu, Shu-Bing Hou, Xian-Ze Sun

**Affiliations:** Department of Spine Surgery, The Third Hospital of Shijiazhuang, Shijiazhuang, China.

**Keywords:** extrapedicular, fracture, kyphoplasty, lumbar, osteoporotic

## Abstract

**Rationale:**

Traditionally, transpedicular approach was used in the treatment of osteoporotic lumbar compression fracture. In order to avoid the risks of pedicle disruption and spinal canal intrusion, extrapedicular approache has been attempted. The aim of the article is to present the modified extrapedicular kyphoplasty technique for the treatment of osteoporotic lumbar compression fracture.

**Patient concerns:**

A 62-year-old woman suffered from severe low back pain after an accidental fall 10 days ago. Low back pain was obvious when turning over and getting out of bed. It was not relieved after bed rest and conservative treatment. Visual analog scale (VAS) of low back pain was 8 points and Oswestry disability index score was 80%.

**Diagnosis:**

Magnetic resonance imaging showed osteoporotic vertebral compression fracture of L2 and L3.

**Interventions:**

We performed modified extrapedicular kyphoplasty for the patient. The technique has a standardized operating procedure. The puncture point of skin is determined according to preoperative computer tomography and X-ray. The puncture point of vertebral body is located at the outer upper edge of the pedicle. The puncture direction is from the upper edge of the pedicle to the lower edge of the contralateral pedicle.

**Outcomes:**

The operation time was 20 minutes. The intraoperative blood loss was 5 mL. The amount of bone cement was 4 mL in L2 and 5 mL in L3. VAS of low back pain was 2 points in 1 day after surgery. Preoperative symptoms were significantly improved.

**Lessons:**

: Modified extrapedicular kyphoplasty is a safe and effective technique for the treatment of osteoporotic lumbar compression fracture, which should be promoted and applied.

## 1 Introduction

With the rapid development of population aging, osteoporosis has become a serious public health problem. Osteoporotic vertebral compression fracture (OVCF) often occurs after minor or no trauma because of reduced bone mineral density.^[1]^ Traditionally, OVCF were treated with bed rest, analgesics, bracing, and physical therapy.^[2]^ Surgical treatment is considered for patients with neurologic deficit or pain refractory to conservative treatment.^[1]^

Percutaneous kyphoplasty (PKP) and percutaneous vertebroplasty (PVP) are minimally invasive treatment options for VCFs, which can provide immediate pain relief, mobility, and an improved quality of life.^[2,3]^ In the last 30 years, the use of PKP and PVP for the treatement of VCFs has been dramatically increased. At the same time, various approach methods are developed to access a fractured vertebral body.^[4]^ Initially, transpedicular percutaneous biopsies were performed for the purpose of histologic analysis of the vertebral body. Then the transpedicular approach was used for PKP and PVP.^[5]^ However, the transpedicular approach may lead to complications of pedicle disruption and spinal canal intrusion.^[1]^ In order to avoid damage to the facet joint and pedicle, extrapedicular approach has been attempted.^[6]^

In 2005, Han et al^[7]^ reported extrapedicular PVP in the treatment of upper and mid-thoracic vertebral compression fracture. In 2007, Ryu et al^[8]^ reported the surgical technique extrapedicular PKP with a single balloon in thoracic vertebral and lumbar vertebrae. In 2011, Cho et al^[4]^ reported extrapedicular PVP and PKP in 74 lumbar vertebrae, confirming the efficacy and feasibility of the extrapedicular approach for lumbar PVP (PKP).

As the lumbar artery (LA) is distributed on the posterolateral side of the vertebral body, the extrapedicular approach has the risk of LA injury.^[10,11]^ Heo et al^[6]^ reported a case of a 73-year old female patient with the left second LA injury following L2 PVP using extrapedicular approach. Biafora et al^[12]^ reported a case of LA injury following percutaneous extrapedicular PKP in an L5 compressed fracture. Selective angiography confirmed intersegmental branch bleeding and this branch originated from the right third LA.

After evaluating the trend and distribution of the LA, Liu L et al^[13]^ suggest the safe puncture area in the vertebral body during extrapedicular PVP (PKP) in lumbar vertebrae should be slightly higher than the sagittal midline of the pedicle. Therefore, we adopt the modified extrapedicular kyphoplasty technique for the treatment of lumbar compression fracture, which with satisfactory result. The puncture point of vertebral body is located at the outer upper edge of the pedicle, which is described below. The patient's informed consent was obtained for publication of this case report. The study obtained ethics committee approval from The Third Hospital of Shijiazhuang.

## 2 Case report

### 2.1 History

A 62-year-old woman suffered from severe low back pain after an accidental fall 10 days ago. Low back pain was obvious when turning over and getting out of bed. It was not relieved after bed rest and conservative treatment. Visual analog scale (VAS) of low back pain was 8 points and Oswestry Disability Index (ODI) score was 80%.

### 2.2 Physical exam

Physical examination showed that the patient with limited lumbar flexion and extension activities. There was severe slamming pain in L2 and L3 spinous process plane but no lower extremity radiation pain. Skin feel and muscle strength of lower lims were normal.

### 2.3 Imaging

**Figure 1:**
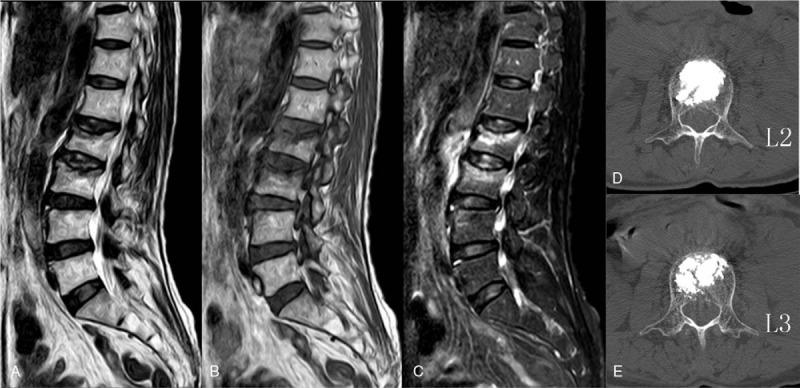
A 62-year-old woman suffered from severe low back pain after an accidental fall 10 d ago. The preoperative MRI (A, B, C) showed osteoporotic vertebral compression fracture of L2 and L3. Postoperative CT (D, E) showed the satisfaction of bone cement was satisfactory. CT = computer tomography, MRI = magnetic resonance imaging.

X-ray, computer tomography (CT) and magnetic resonance imaging showed OVCF of L2 and L3 (Fig. 1). The bone density T value was -4.8. OVCF and postmenopausal osteoporosis were diagnosed.

### 2.4 Operation

**Figure 2:**
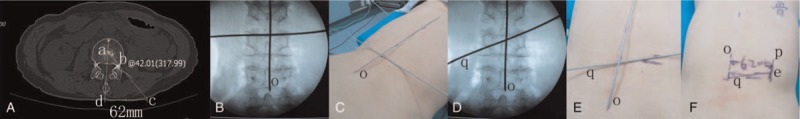
The distance of skin puncture point and puncture angle were calculated according to preoperative CT (A). The midpoint of vertebral body was point a and the outer upper edge of pedicle was point b. The line connecting point a and point b was m. The intersection of line m and the skin was point c. The midline of the vertebral body was n. The intersection of line n and the skin was point d. The distance between point c and d was 62 mm. The angle between line m and n was 42°. The midline of the spinous process was marked as line o. Parallel line p was marked with 62 mm on the right side of line o. The line connecting the upper edge of the right pedicle to the lower edge of the left pedicle was marked as q. The intersection of q and p was marked as point e which was the puncture point of skin (B, C, D, E, F). CT = computer tomography.

We performed modified extrapedicular kyphoplasty for the patient. The distance of skin puncture point and puncture angle were calculated according to preoperative CT (Fig. 2). The midpoint of vertebral body was point a and the outer upper edge of pedicle was point b. The line connecting point a and point b was m. The intersection of line m and the skin was point c. The midline of the vertebral body was n. The intersection of line n and the skin was point d. The distance between point c and d was 62 mm. The angle between line m and n was 42°. So the distance of skin puncture point to the midline of the spinous process was 62 mm. Outreach angle of puncture needle was 42°.

**Figure 3:**
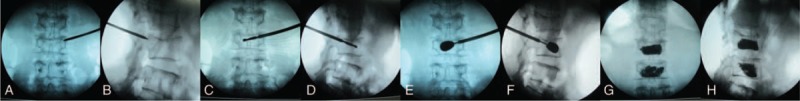
The entry point of vertebral body was outer upper edge of pedicle in AP view (A) and upper edge of pedicle in lateral view (B). The puncture direction was from the upper edge of pedicle to the lower edge of contralateral pedicle. Outreach angle of puncture needle was 42°. A drill was used through the tube. The ideal position of drill tip should cross the spinous process in AP view (C) and cross the center of vertebral body in lateral view (D). The balloon was used to restore vertebral height (E, F). Finally, bone cement was injected. The same technique was performed in L3 and L2 (G, H). AP = anteroposterior.

The patient was placed in a prone position. An image of the vertebral body was adjusted under fluoroscopy with anteroposterior (AP) and lateral views (Fig. 3). The midline of the spinous process was marked as line o. Parallel line p was marked with 62 mm on the right side of line o. The line connecting the upper edge of the right pedicle to the lower edge of the left pedicle was marked as q. The intersection of q and p was marked as point e which was the puncture point of skin. Local anesthesia with 1% lidocaine. A puncture needle was used to probe the entry point. The entry point of vertebral body was outer upper edge of pedicle in AP view and upper edge of pedicle in lateral view. The puncture direction was from the upper edge of pedicle to the lower edge of contralateral pedicle. Outreach angle of puncture needle was 42°. It was replaced with a tube when the needle entered vertebral body.

A drill was used through the tube. The ideal position of drill tip should cross the spinous process in AP view and cross the center of vertebral body in lateral view. The balloon was used to restore vertebral height. Finally, bone cement was injected. The same technique was performed in L3 and L2. The amount of bone cement was 4 mL in L2 and 5 mL in L3. The distribution of bone cement was satisfactory and without bone cement leakage. The operation time was 20 minutes. The intraoperative blood loss was 5 mL.

### 2.5 Post-operative course

After the procedure, the patient was placed in the supine position and asked to remain flat for at least 2 hours. The patient was encouraged to ambulate from the next morning.

### 2.6 Follow-up/imaging

VAS of low back pain was 2 points in 1 day after surgery. VAS of low back pain was zero point and ODI score was 10% in 12 months after surgery. Preoperative symptoms were significantly improved. Postoperative CT showed the satisfaction of bone cement was satisfactory.

## 3 Discussion

Percutaneous PKP and PVP are minimally invasive treatment options for VCFs, which can provide immediate pain relief, mobility, and an improved quality of life by injecting bone cement into the body. However, PVP involves the uncontrolled high-pressure injection of low viscosity of cement into the collapsed body which has the potential risk of bone cement leakage. On the other hand, PVP has a limitation in restoring a compressed vertebral body. PKP usually involves injection of high-viscosity cement into the cave created by the balloons under low pressure which has lower risk of bone cement leakage. In addition, PKP can partially restore the vertebral height.

The current standard technique for PKP involves bipedicular approaches. However, unipedicular approach (either intrapedicular or extrapedicular) can reduce the risk associated with the cannulation of both pedicles and also reduce operative time, radiation exposure, and costs. Steinmann et al^[14]^ performed a biomechanical cadaver study which showed that unipedicular PKP is comparable with bipedicular PKP in the restoration of vertebral body strength, stiffness, and height. Kim AK et al^[15]^ reported that unipedicular and bipedicular approaches have no significant difference in clinical outcome. So unipedicular PKP is a safe, efficacious, faster, less expensive, and less radiation exposure technique.^[2]^

Unipedicular PKP include transpedicular and extrapedicular approaches. However, transpedicular approach has higher risks of pedicle disruption and spinal canal intrusion compared with extrapedicular approach. In addition, the cement injection needle can reach the middle anterior portion of the vertebral body more easily with the extrapedicular technique. Therefore, extrapedicular approach is gradually be used in unipedicular PKP.

However, literature have reported LA injury following percutaneous extrapedicular PKP. As the LA is distributed on the posterolateral side of the vertebral body, the extrapedicular approach has the risk of LA injury. To avoid injury to the LA, we adopt the modified extrapedicular kyphoplasty technique for the treatment of lumbar compression fracture.

There were several technical points in modified extrapedicular kyphoplasty. First, the puncture point of skin is determined according to preoperative CT and X-ray, which makes the operation more precise. Second, the entry point of vertebral body was outer upper edge of pedicle in AP view and upper edge of pedicle in lateral view, which effectively avoid injury to segmental arteries. Third, the puncture direction is from the upper edge of the pedicle to the lower edge of the contralateral pedicle, which reaching the midline of the vertebral body with a single cannula. So, it can reduce operative time, radiation exposure, and costs. Finally, extrapedicular approach puncture can maintain the integrity of the pedicle cortex, avoid complications due to transpedicular approach, preserve the axial or lateral biomechanical stability of the spine and provide a choice for patients with pedicle dysplasia.^[9]^

However, in some lumbar vertebrae without LAs, there was an intersegmental branch passes through the lateral side of the pedicle that comes from the upper segmental LA and it mostly appeared in L4 and L5, especially in female patients.^[13]^ Thus, modified extrapedicular kyphoplasty was not suggested for use in L4 and L5, especially in female patients.

## 4 Conclusion

Modified extrapedicular kyphoplasty is a safe and effective technique, which should be promoted and applied. The technique has a standardized operating procedure. The puncture point of skin is determined according to preoperative CT and X-ray. The puncture point of vertebral body is located at the outer upper edge of the pedicle. The puncture direction is from the upper edge of the pedicle to the lower edge of the contralateral pedicle. However, it was not suggested for use in L4 and L5, especially in female patients.

## Author contributions

**Conceptualization:** Li-Min Wang, Feng-Yu Liu, Xian-Ze Sun.

**Data curation:** Li-Min Wang, Feng-Yu Liu.

**Formal analysis:** Shu-Bing Hou.

**Investigation:** Kuan Lu, Zhao Liu.

**Methodology:** Feng-Yu Liu, Xian-Ze Sun.

**Resources:** Zhao Liu.

**Supervision:** Shu-Bing Hou.

**Writing – original draft:** Feng-Yu Liu.

**Writing – review & editing:** Li-Min Wang, Kuan Lu, Xian-Ze Sun.
